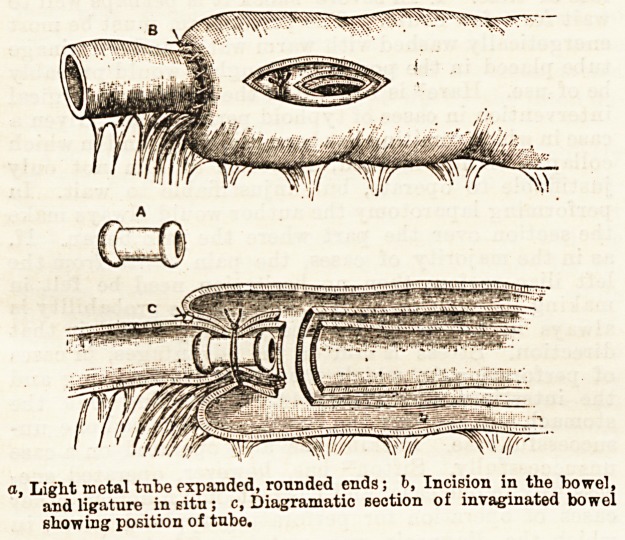# Surgery of the Intestines

**Published:** 1895-09-21

**Authors:** 


### SURGERY OF THE INTESTINES.

**Intestinal Obstruction.**—Mayo Robson[1] classifies intestinal obstruction depending on gall stones as follows: (1) The form dependent on local peritonitis in the region of the gall bladder leading to paralysis of the bowel. Two cases of this class both yielded to general treatment without operation. (2) Volvulus of the small intestine dependent either on the violence of the colic caused by an attack of cholelithiasis, or on the contortions induced by the passage of a large concretion through the small intestine. In two cases of this class the author has performed laparotomy and untwisted the volvulus, recovery following in each case. (3) Mechanical obstruction due to the passage of a large concretion through the small intestine. In two cases of this class enterotomy was performed, with removal of the concretion, both cases recovering. (4) Obstruction depending on adhesions or on stricture, the result of past gall-stone attacks or of healing fistulæ. The third form described is the commonest. Needling a stone through the already damaged intestine should be avoided, a small incision being much more satisfactory. In Robson's experience gallstones in the intestine are seldom palpable through the abdominal wall, owing to the accompanying distension. Removing the stone by enterotomy is preferable to pushing it on into the colon; and unless the bowel is considerably damaged, it is not necessary to fix it near the wound. Taylor[2] records the successful removal of a gallstone weighing one and a-quarter ounce, which caused obstruction in a woman aged fifty-six years. Smith[3] and Kinneir[4] both publish unsuccessful cases of a similar nature. Thornley Stokes[5] deprecates the use of O'Beirne's tube for the administration of enemata in cases of obstruction. He finds it best to use for this purpose an ordinary red-rubber tube four-eighths to five-eighths of an inch in diameter, such as is used for washing out the stomach, and having a large glass or celluloid funnel fitted to the dilated extremity. It is convenient to have circles marked at three, six, and nine inches from the end of the tube, so as to know what length of it lies in the rectum. The patient lies on his back or left side, with the pelvis raised, so as to facilitate the passage of fluid into the sigmoid and descending colon. Eight or ten gallons of water may be employed at one sitting. The surgeon sits on the right of the patient's bed, and introduces the end of the tube into the rectum as far as may be desired. An assistant pours water into the funnel, and the pressure may be varied and adjusted by the height to which it is raised by the operator. When as much water has been introduced as can be borne, the funnel is removed, that end of the tube lowered to a basin placed on the floor, and the fluid allowed to run out of the bowel. By repeated operation of this kind large quantities of water may be used, and the bowel emptied without the patient being exhausted by straining or by the necessity of changing his position. The solution of fæces and expulsion of flatus are assisted by pushing the tube in and out, and by the varying hydrostatic pressure, caused by alternately raising and lowering the funnel at the free end of the tube. Jacobson[6] draws attention to the great mortality attending operations for intestinal obstruction. A large proportion of cases must be fatal, partly on account of the vulnerability of the parts and the attendant collapse, and partly on account of the failure of operation to detect or relieve the seat of strangulation. Amongst the most favourable cases for operation are those of obstruction by bands and those in which acute obstruction supervenes upon an unsuspected chronic obstruction. Enemata by the long tube are useless for severe, acute obstruction. Every case should be submitted to operation if the patient's condition warrants it, and provided the surgeon knows how far it is safe to go. No operation should be unduly prolonged; if the obstruction is not readily found, it is better to open a distended coil, insert a Paul's tube and close the abdomen. Benham[7] relates a somewhat rare case of successful reduction of a volvulus of the sigmoid flexure causing intestinal obstruction.

**Intussusception.**—When collapse and gangrene are both present in these cases Paul[8] thinks the best chance is afforded by bringing the resected ends out of the abdomen and approximating them subsequently. In many patients the condition is less grave, and in these an operation on the lines first suggested by Barker[9] seems to be the best. When the invagination is short and easily brought out through the incision in the outermost layer of the intussusception, Barker's operation perhaps leaves nothing to be desired; but when the invagination is too extensive for this, and has to be divided *in situ*, the operation certainly has dangers from hæmorrhage and from the tendency which the large stump of mesentery has to retract when the weight is taken off, so that the preliminary sutures are liable to tear out. Paul therefore proposes that the surgeon should be armed with a short metal tube made of aluminium and shaped somewhat as in the engraving (Fig. A). The preliminary sutures—very few of which are required—connecting the intussusception with the intussuscipiens and the first incisions are made as represented by Mr. Barker, then the returning and entering layers are also respectively incised (see Fig. B), and the tube, grasped with forceps, is pushed into the position as shown in Fig. C. A stout silk ligature is now made to surround the intussusceptum just above the incisions in it, and is tightly tied. Finally, the intussusceptum is cut off below the liga-

*a*, Light metal tube expanded, rounded ends; *b*, Incision in the bowel, and ligature in situ; *c*, Diagramatic section of invaginated bowel showing position of tube.

ture and withdrawn, and the wound in the outer layer is closed in the usual way. The end aimed at is exactly the same as that attained by other methods, but the writer thinks it possesses the following advantages: (1) A saving of time, as the ligature replaces many sutures, and also closes the blood-vessels before they are divided; (2) the operation is practically bloodless; (3) disengagement of the invaginated stump is impossible, as it is firmly held by the ligature for at least three days. Roughton[10] and Pick[11] both report successful cases of laparotomy for intussusception. In Roughton's case the infant was only four months old.

**Tumours of the Ileo-Cæcal Region** are mostly in the form of carcinomata and local intestinal tuberculosis. Körte[12] has had thirteen cases, seven of which were operated on successfully; two of these, however, ended fatally in eight and eleven months later respectively. The commencement, in the case of malignant disease, is very insidious. Generally the symptoms are those of typhlitis and coprostasis. Suppuration frequently takes place in the neigbourhood of the bowel, and it is often difficult to distinguish such abscesses from those resulting from perityphlitis. The diagnosis is frequently only made with the microscope. As regards treatment, resection and union of the divided parts are necessary. Symptoms of acute obstruction are contra-indications for resection and suture. Simple enterostomy is here called for, with resection later on. The extent of the tumour may be prohibitory of operation, as also is advanced cachexia. Of these tuberculous cases two were hereditarily predisposed. Abbe[13] reports an unsuccessful operation on a case of sarcoma or carcinoma of the caput coli, and Pilliet and Thiery[14] found on opening the abdomen that a large tubercular mass in the cæcum was irremovable, the patient dying fifteen days afterwards of general tuberculosis.

**Intestinal Resection and Anastomosis.**—König,[15] whilst recognising the fact that modern methods of establishing intestinal anastomosis by bone-plates, metal buttons, &c., favour rapidity of operation, holds that a prolonged laparotomy does not lead to shock. For this reason he is not disposed to substitute for older and safe operations, which may take some time in their performance, rapid methods, the safety of which seems to him to be doubtful. That the use of Murphy's button may enable inexperienced surgeons to perform these operations is regarded as being, as far as the patients are concerned, rather a disadvantage than an indication of advance. Dawbarn[16] discusses the relative value of the Murphy button and absorbable plates in intestinal anastomosis. With the exception of the operation of cholecystenterostomy he maintains that approximation by Murphy's button gives inferior results to those obtained by such absorbable plates. He quotes several unsuccessful cases, where the Murphy button was used, to support his argument, and also refers to Magill's article[17] in which the statistics are discussed in detail. With reference to the best form of absorbable plate, Dawbarn maintains that the use of raw vegetable plates is somewhat of an improvement upon those made of decalcified bone, though, of course, the principle remains the same. This is true for the following reasons: 1. The vegetable plates are always obtainable at a minute's notice. 2. They cost practically nothing. 3. They are easily made by anybody with a penknife. 4. They can without difficuly be made to have a very long opening—often a desideratum—to guard against stenosis from late contraction. Indeed, with sweet potatoes, it is easy to cut a plate that shall have a four-inch opening. 5. They are softened, absorbed, and hence gotten out of the way sooner than bone plates, which is a great advantage over the bone plates. Indeed, the latter plates, taking a week or so to absorb, have in one or two instances finally caused death by sliding on each other, after a number of days, enough to obstruct the opening. 6. They are more pliable than bone, easier to preserve, and better brought together in making the anastomosis, slipping much less. Though absorbed in from a day to two or three days, depending on what portion of the alimentary canal they occupy, there is no reported case where they have been absorbed too soon. The most rigid test of this point is, of course, a gastro-enterostomy; and Von Baracz's repeatedly published successes with such plates, in this operation, speak for themselves. Treves,[18] on the other hand, speaks highly of the Murphy button, and

Murphy[19] himself still maintains the superiority of his device over all other, and supports his argument by the following remarkable statistics[20] of recorded cases in which the button was used:—

| | Cases. | Deaths. |
|---|---|---|
| Gastro-enterostomy for malignant disease ... | 29 ... | 9 |
| Pylorectomy ... ... ... ... ... | 4 ... | 1 |
| Cholecystduodenostomy ... ... ... ... | 38 ... | 1 |
| Cholecystenterostomy for malignant disease ... | 8 ... | 7 |
| Resection of bowel for internal obstruction ... | 14 ... | 1 |
| ,, ,, ,, gangrenous hernia ... | 12 ... | 2 |
| ,, ,, ,, fæcal fistula ... ... | 9 ... | 0 |
| ,, ,, ,, non-malignant disease | 48 in all. | 3 |
| ,, ,, ,, malignant disease ... | 30 ... | 7 |
| Lateral approximation— 12 cases } 5 for malignant disease, with | | 2 |
| 7 for benign growths ... | | 0 |

Murphy thinks it is wrong to operate or to use the button in cases of malignant disease of the gall bladder. Many cases are reported of the use of the Murphy button, as examples of which we may refer to those of Lane,[21] Morton,[22] Bush,[23] Banks[24] and Murphy[25] (Sunderland). In Lane's case of gastro-enterostomy the button had been retained for 160 days, and had not come away at the time of reporting of the case, but the intestinal tract had shown no signs of irritation.

**Jejunostomy** has been performed in five cases by Hahn,[26] who suggests the following as proper indications for this operation: 1. When death is threatening in cases of severe corrosion of the stomach and œsophagus by a caustic acid or alkali. 2. In cases of carcinoma of the œsophagus and stomach, in which a bougie cannot be passed, and gastrostomy is contra-indicated by contraction of the stomach. 3. In cases of carcinoma of the pylorus, in which the stomach is so involved that neither gastro-enterostomy nor resection can be performed.

**Treatment of Artificial Anus.** — Chaput[27] records thirty-five cases of stercoral fistulæ and artificial anus which he has treated, and has formulated the following conclusions. He says artificial anus can be dealt with by four different methods: 1. By the application of enterotomy, followed by obliteration of the fistula. (*a*) Enterotomy is indicated when the cases are uncomplicated and the aperture is easily accessible, together with a thin and long partition. When the spur is long and thin, section between two pairs of forceps and immediate suture (Richelot's method) is best; but when the spur is long and somewhat thickened, it is better to make the suture between two long pairs of forceps, which are allowed to remain in position. (*b*) After destruction of the spur the stercoral fistula is closed. Small fistulæ are closed by lateral enterorraphy, during which operation the margins of the fistula are freely separated from their surroundings, and then united with two tiers of sutures, the peritoneum being opened or not, according to the requirements of each individual case. In large fistulæ lateral enterorraphy may be employed, but the peritoneum should not be opened. 2. Resection. This, as a rule, is contra-indicated, but may be necessary when the intestine is easily friable and largely lacerated in the course of a lateral enterorraphy. 3. Longitudinal enterorraphy. This is sometimes advisable when enterotomy for some reason or other is contra-indicated. A circular incision is made in the skin around the artificial anus, and opening the peritoneal cavity. The two ends are drawn out, a longitudinal slit is made in each, and then the margins of the slits of the same side are sewn together with sutures. 4. Entero-anastomosis, followed by ligature of the two ends between the points of anastomosis and the stercoral aperture. This is indicated when the intestine is very friable at the seat of the artificial anus, or when there is a considerable constriction of the bowel in the neighbourhood of the external aperture, and also when the inferior end is obliterated at the level of the artificial anus. Smith[28] also speaks highly of an extra-peritoneal operation somewhat similar to one of the methods of Chaput described above.

**Perforating Ulcers of the Intestine.**—Sheild,[29] in publishing two cases of duodenal ulcer upon which he has operated, draws attention to the following points: 1. That in perforative peritonitis there is nothing to point to the duodenum as the site of lesion unless it is clearly made out that the onset of pain was in the epigastrium or right hypochondrium; or that previous epigastric symptoms, as pain and vomiting, had occurred. 2. That, considering the frequency of duodenal ulcer in males, the possibility of this affection should always enter the minds of surgeons who are called to a case of perforative peritonitis in a man. 3. That non-feculent and sometimes acid nature of the extravasated fluids and gas may serve as a most important diagnostic aid, and the incision may be made small as an exploratory effort, only until this vital point is made clear. When once the surgeon has made up his mind on this point, the region of the stomach and duodenum should be explored without loss of time. 4. In severe shock it is perhaps well to wait for a few hours. The peritoneum must be most energetically washed with warm water, and a drainage tube placed in the pouch of Douglas would probably be of use. Hare[30] is hopeful of the results of surgical intervention in cases of typhoid perforation. Given a case in which vomiting has been inhibited, and in which collapse is not marked, it seems to him not only justifiable to operate, but unjustifiable to wait. In performing laparotomy the author would always make the section over the part where the pain began. If, as in the majority of cases, the pain starts from the left iliac region, then no hesitation need be felt in making the incision on that side, as the probability is always in favour of the lesion being found in that direction. Stress is laid on the advantages, in cases of perforation in which there is much vomiting and the intestines are distended, of washing out the stomach and incising the bowel. He has had one unsuccessful case. Parkin[31] has also operated on a case unsuccessfully. Sifton[32] has, however, operated successfully in one case, and has collected nineteen other cases of operation for perforating typhoid ulcer, in which the diagnosis was certain. Of these twenty cases there were four recoveries, which is a much better result than by the expectant plan of treatment, under which recovery rarely takes place.

[1] Brit. Med. Journ., Jan. 26th, 1895, p. 194. [2] Lancet, April 6th, 1895, p. 867. [3] Med. Chron., April, 1895. [4] Brit. Med. Journ., March 9th, 1895, p. 529. [5] Dublin Journ. Med. Sci., Feb. 1st, 1895. [6] Brit. Med. Journ., April 13th, 1895. [7] Ibidem, May 4th, 1895. [8] Lancet, March 30th, 1895. [9] Lancet, Jan. 9th, 1892. [10] Lancet, Feb. 23rd, 1895. [11] Lancet, March 23rd, 1895. [12] New York Med. Rec., April 20th, 1895. [13] Annals of Surg., Vol. I., 1895, p. 592. [14] Practitioner, Feb., 1895, p. 183. [15] Brit Med. Journ., Feb. 16th, 1895. [16] Annals of Surgery, Feb., 1895, p. 166. [17] Ibidem, Sept., 1894. [18] Practitioner, June, 1895, p. 481. [19] Lancet, April 27th, 1895. [20] Med. News, Feb. 9th, 1895, and Practitioner, May, 1895. [21] Lancet, May 4th, 1895. [22] Brit. Med. Journ., April 20th, 1895. [23] Lancet, April 6th, 1895. [24] Brit. Med. Journ., Feb. 23rd, 1895. [25] Ibidem, April 20th, 1895. [26] Ibidem, Feb. 9th, 1895. [27] Med. Rec., April 20th, 1895. [28] Bristol Med. Chi. Journ., March, 1895. [29] Internat. Med. Mag., Jan., 1895, and Lancet, May 11th, 1895. [30] Intercol. Quart. Journ. of Med. and Surg., Feb., 1895, and Brit. Med. Journ., April 20th, 1895. [31] Brit. Med. Journ., Jan. 26th, 1895. [32] Chicago Clin. Review, April, 1895.

## Figures and Tables

**Figure f1:**